# Relationship between COVID-19 and “three inflammations and one deafness”: a systematic review and meta-analysis

**DOI:** 10.3389/fimmu.2026.1690788

**Published:** 2026-02-24

**Authors:** Lisha Liu, Hong Zhao, Jing Qiao, Nai Liu, Wei Tao, Shengli Wei

**Affiliations:** 1 Department of Otolaryngology, First Teaching Hospital of Tianjin University of Traditional Chinese Medicine, Tianjin, China; 2 Department of Otolaryngology, National Clinical Research Center for Chinese Medicine, Tianjin, China

**Keywords:** allergic rhinitis, COVID-19, meta-analysis, otitis media, pharyngitis, tinnitus and deafness

## Abstract

**Background:**

The relationship between “three inflammations and one deafness” (allergic rhinitis, pharyngitis, otitis media, tinnitus, and deafness) and coronavirus disease 2019 (COVID-19) is currently unclear, and this study aims to investigate their correlation.

**Methods:**

We searched the relevant literature in three databases (Embase, Cochrane Library, and PubMed) from their inception through July 2024, and the investigator strictly reviewed the literature according to the screening criteria to determine the included studies. We extracted relevant data information and conducted quality assessment and meta-analysis.

**Results:**

From 5,950 records screened, five cohort studies were included. The pooled analysis using a random-effects model showed no statistically significant association between COVID-19 and “three inflammations and one deafness” (OR = 1.03, 95% CI: 0.85–1.26, *P* = 0.74), with substantial heterogeneity (*I*² = 89%, *P* < 0.001). Critically, subgroup analyses revealed that the diagnostic criteria for “three inflammations and one deafness” were a key source of this heterogeneity. A significant association was observed in studies using physician-diagnosed outcomes (OR = 1.30, 95% CI: 1.08–1.56, *P* = 0.006, *I*² = 0%), whereas no significant association was found in studies based on self-reported symptoms (OR = 0.89, 95% CI: 0.69–1.15, *P* = 0.38, *I*² = 96%). Analyses by specific conditions yielded mixed results: No significant association was observed for hearing loss (OR = 0.93, 95% CI: 0.69–1.25, *P* = 0.62). For allergic rhinitis (OR = 1.19, 95% CI: 0.47–3.02, *P* = 0.71) and tinnitus (OR = 1.11, 95% CI: 0.88–1.39, *P* = 0.38), the point estimates suggested potential positive trends, but the associations were not statistically significant, and confidence intervals were wide. Subgroup analyses by some regions and COVID-19 diagnostic criteria did not reveal consistent significant associations.

**Conclusions:**

This meta-analysis found no consistent association between COVID-19 and “three inflammations and one deafness,” primarily due to significant heterogeneity. Evidence suggests a link between COVID-19 and physician-diagnosed “three inflammations and one deafness,” which strongly depends on the rigor of outcome assessment, highlighting the need for standardized clinical diagnoses in future research.

**Systematic Review Registration:**

https://www.crd.york.ac.uk/PROSPERO/, identifier CRD42023438076.

## Introduction

Since its outbreak, the coronavirus disease 2019 (COVID-19) has become a significant global health burden. Although the World Health Organization (WHO) declared the end of its pandemic emergency phase in 2023, COVID-19 remains a persistently prevalent respiratory infectious disease, with its cumulative disease burden still enormous. As of July 2024, more than 775 million people have been diagnosed with COVID-19 worldwide, and 7.05 million people have now died. Among them, more than 90 million people have been diagnosed in China, and more than 120,000 people have died from the disease (1). In addition to respiratory symptoms such as fever and cough, COVID-19 also has clinical manifestations of systemic symptoms such as diarrhea, arrhythmia, loss of appetite, nausea, and vomiting (2).

“Three inflammations and one deafness” primarily refers to immune-inflammatory diseases related to the ear, nose, and throat, including allergic rhinitis (AR), pharyngitis, otitis media, tinnitus, and hearing loss. Typically, viral infections and allergen exposure act as initial triggers, activating the immune system in the nasal and pharyngeal mucosa, which leads to local inflammation and subsequently causes rhinitis and pharyngitis. This inflammation can spread through anatomical connections or inflammatory mediators, affecting the middle ear and resulting in otitis media. Simultaneously, these cascading immune-inflammatory reactions may lead to long-term or severe complications, such as tinnitus and hearing loss. Among them, AR has a global incidence rate of 10%–40% (3). Additionally, the number of patients with tinnitus has increased year by year, and it is reported that over 93% of patients experience accompanying or resulting hearing loss (4). Tinnitus occurs in as many as 60%–90% of patients with acquired deafness (5). Therefore, “three inflammations and one deafness” is not only a common condition in otolaryngology but also a widely recognized global health issue.

At present, the association of COVID-19 and “three inflammations and one deafness” is still controversial. It was reported that AR might be a risk factor for long-term COVID-19 and might contribute to its increased risk (6). One study showed that symptoms such as tinnitus and hearing loss were positively associated with typical symptoms of severe acute respiratory syndrome coronavirus 2 (SARS-CoV-2) infection. The risk of ear symptoms increased with increasing symptoms of SARS-CoV-2 infection (7). Another study found that damage to the inner ear and auditory pathways was associated with COVID-19 through objective and behavioral audiometry (8). However, some studies reported that COVID-19 had a lower incidence rate in AR patients, and its prognosis was similar to that of non-AR patients. The incidence of AR in COVID-19 was also lower (9). Therefore, the association between COVID-19 and “three inflammations and one deafness” is currently unclear. This article aims to analyze the relevant evidence based on existing analytical research, which is to determine the correlation between them and provide the scientific basis for disease prevention and treatment.

## Methods

### Data sources and search strategy

We conducted the evaluation and analysis based on the preferred reporting items (10) and registered on PROSPERO (CRD42023438076). We searched the relevant databases (Embase, Cochrane Library, and PubMed) for articles published since their establishment until July 2024. The main terms we searched for include COVID-19 terms (“COVID 19,” “2019-nCoV Infection,” “SARS-CoV-2 Infection,” etc.) and “three inflammations and one deafness” terms (“rhinitis,” “nasal catarrh,” “pharyngitis,” “pharyngitides,” “otitis media,” “middle ear inflammation,” “tinnitus,” “tinnitus, tensor palatini induced,” “deafness,” “complete hearing loss,” etc.) and other terms (“cohort,” “case control,” etc.). Furthermore, we discussed with experts to get more information from the references of the article.

### Study selection and eligibility criteria

The two researchers screened the articles by the established criteria and contacted the relevant authors for more information. If a disagreement could not be resolved, a third investigator would evaluate it. The inclusion criteria included the following: 1) study population: patients diagnosed with COVID-19; 2) comparison of the control group; 3) outcomes: new onset or occurrence of “three inflammations and one deafness,” along with reporting of related effect measures (such as odds ratio or relative rate, etc.); 4) study design: analytical studies, including cohort studies and case–control studies; 5) articles published in English; and 6) scientific diagnostic criteria for diseases. The exclusion criteria were as follows: 1) meeting reports, letters, reviews, case reports, animal experiments, and narrative comments; 2) full text and repeatedly published articles not available; 3) the topic or results not relevant to the study; and 4) no research designs of interest.

### Data extraction and quality assessment

After reading the articles that met the standards, researchers extracted information such as year, country, number of participants, age, and sample source. Meanwhile, another researcher evaluated the accuracy. A third investigator assisted with the evaluation in case of disagreement. Quality score was performed by the Newcastle–Ottawa Scale (NOS), and studies with a score of 7 or above were considered high quality (11).

### Data synthesis

We performed data extraction of the included studies, used inverse variance, and tested for heterogeneity using *I*^2^. When *I*^2^ ≥50%, the random-effects model was mainly used. When *I*^2^ <50% (low heterogeneity, the fixed-effects model was mainly used. The heterogeneity was judged by subgroup analysis, and the outcome stability was determined by sensitivity analysis. The software used was Review Manager 5.4.

## Results

### Database search results

**Figure 1:**
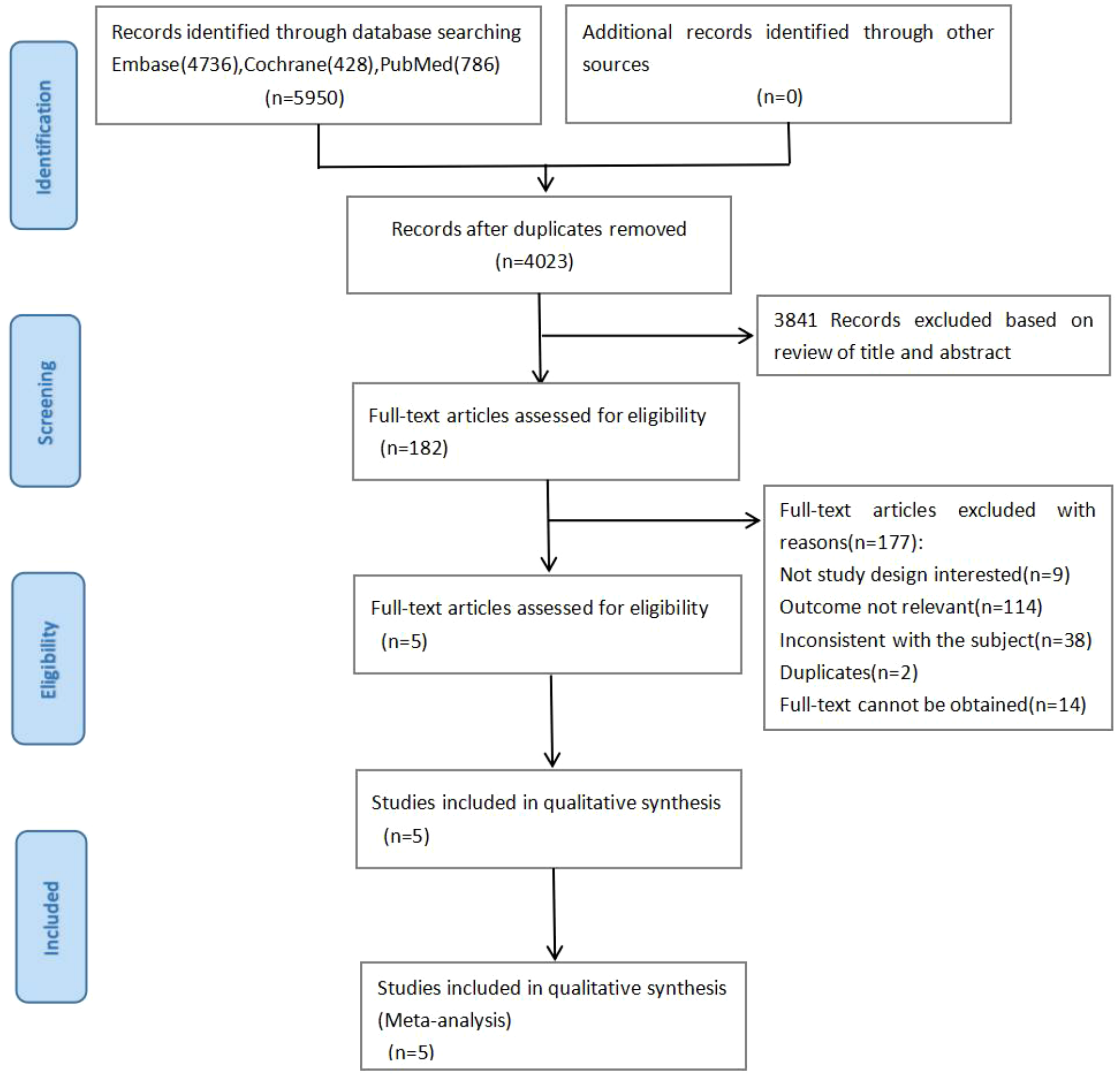
Flowchart of literature screening.

We retrieved a total of 5,950 articles (including Embase 4,736, Cochrane 428, and PubMed 786). Among these, 1,927 duplicate articles were excluded. After reviewing titles and abstracts, 3,841 articles were excluded, leaving 182 articles for full-text review. Ultimately, 5 studies were included through reading and screening (**Figure 1**).

### Study characteristics

We include five cohort studies from 2021 to 2024 with a total of more than 460,000 participants from around the world (mainly in Europe). Patients have a wide age coverage. The diagnostic criteria for COVID-19 are a positive test (the technologies vary by country and research, such as SARS-CoV-2 nucleic acid testing, pathogen antibody testing, multistep antibody testing, immunological testing, etc.), physician diagnosis, and self-report. “Three inflammations and one deafness” is based mainly on physician diagnosis and self-report (**Table 1**).

**Table 1:** Characteristics of the five studies.

Author	Year	Country	Participants (*N*)	Age (years)	Study design	Source of case samples	Criteria of COVID-19	Criteria of inflammations and deafness
Wang et al. (12)	2024	China	468	>18	Cohort study	TCC	Positive test and self-report	Self-report
Africa et al. (13)	2023	Globality	375,171	18–90	Cohort study	TriNetX	Positive test and physician diagnosis	Physician diagnosis
Ren et al. (14)	2022	The United Kingdom	70,557	40–69	Cohort study	UKB	Positive test and physician diagnosis	Self-report
Kostev et al. (15)	2022	Germany	6,568	<18	Cohort study	IQVIA	Physician diagnosis	Physician diagnosis
Stacevičienė et al. (16)	2021	Lithuania	9,238	<18	Cohort study	VUHSK	Positive test and physician diagnosis	Physician diagnosis

TCC, three communities in Chengdu, China; TriNetX, Global Federated Research Network; UKB, UK Biobank; IQVIA, Disease Analyzer Database; VUHSK, Vilnius University Hospital Santaros Klinikos.

### Quality assessment

According to the scoring criteria of cohort studies in NOS, we scored eight items involved in the five studies, including the selection of exposed and non-exposed groups and the adequacy of follow-up. The results showed that four studies were of high quality (total score ≥ 7) (**Table 2**).

**Table 2:** Quality evaluation according to the NOS.

Study	Selection				Comparability	Outcome			Quality score
	Exposed groups	Unexposed groups	Ascertainment of exposure	Without outcome of interest	Comparability	Evaluation	Enough time	Adequacy	
Wang et al. (12)	★	★	★	★	★★	★	☆	★	8
Africa et al. (13)	★	★	★	★	★★	★	☆	★	8
Ren et al. (14)	★	★	★	★	★☆	★	☆	☆	6
Kostev et al. (15)	★	★	★	★	★★	★	★	★	9
Stacevičienė et al. (16)	★	★	★	★	★★	★	☆	☆	7

★: The score is 1; ☆: the score is 0.

### The association between COVID-19 and “three inflammations and one deafness”

**Figure 2:**
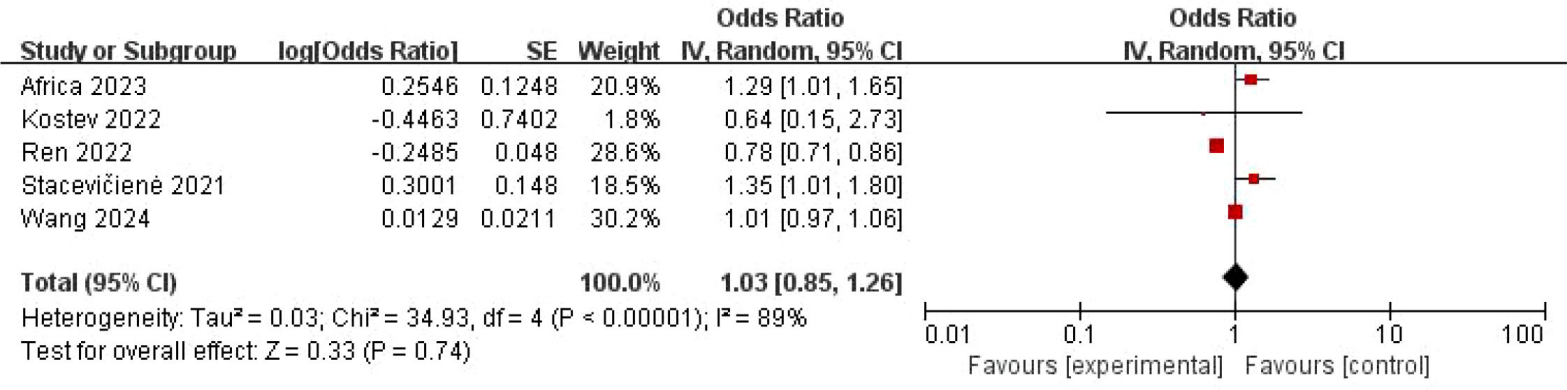
Forest map of the relationship between “three inflammations and one deafness” and COVID-19.

**Figure 3:**
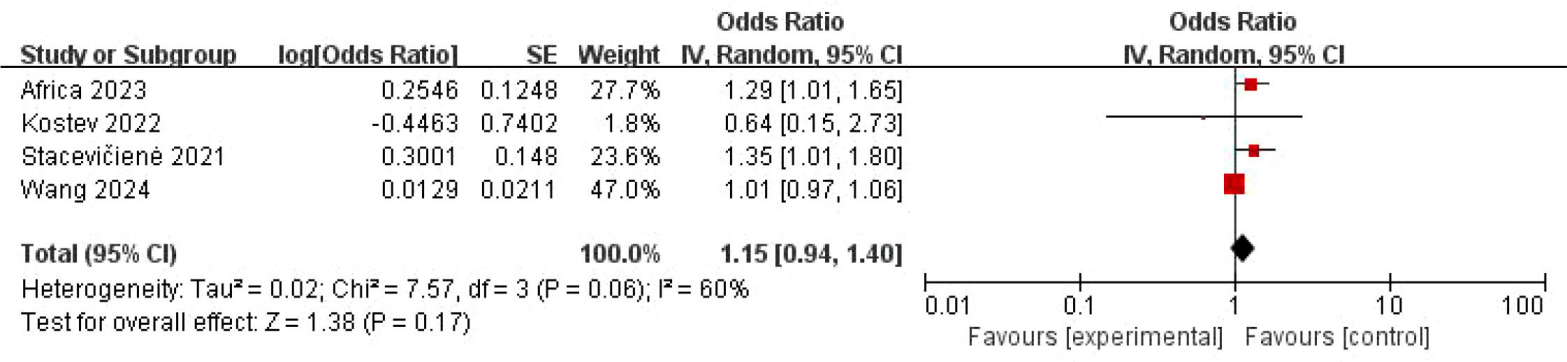
Forest map of the relationship between “three inflammations and one deafness” and COVID-19—only relatively high-quality studies were included.

**Figure 4:**
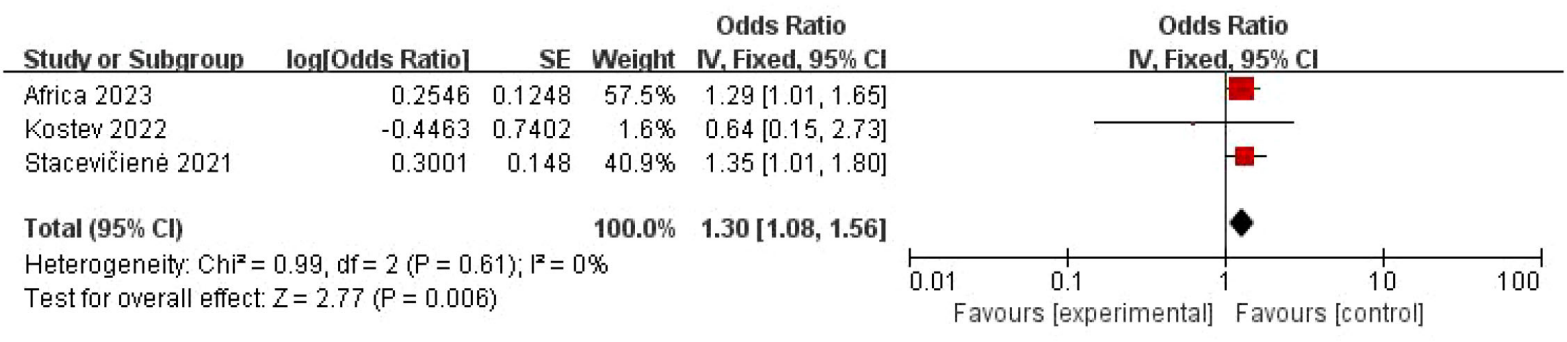
Forest map of the relationship between “three inflammations and one deafness” and COVID-19—only the physician diagnostic criteria were included.

The pooled analysis using a random-effects model showed no statistically significant association between COVID-19 and “three inflammations and one deafness” (OR = 1.03, 95% CI: 0.85–1.26, *P* = 0.74) (**Figure 2**). However, substantial heterogeneity was observed (*I*² = 89%, *P* < 0.001), suggesting potential effect modifiers. To investigate the sources of heterogeneity, we performed sensitivity analyses. First, excluding one study (Ren et al., 2022) with a markedly different effect size and methodological concerns marginally reduced heterogeneity and increased the point estimate, though the association remained non-significant (OR = 1.15, 95% CI: 0.94–1.40, *P* = 0.17; *I*² = 60%, *P* = 0.06) (**Figure 3**). Given the clinical plausibility that diagnostic rigor could be a key moderator, we then conducted a pre-specified subgroup analysis based on the diagnostic criteria for “three inflammations and one deafness.” This analysis yielded a decisive finding: in the subgroup of studies using physician-confirmed diagnoses, a statistically significant and homogeneous association was observed (OR = 1.30, 95% CI: 1.08–1.56, *P* = 0.006; *I*² = 0%) (**Figure 4**). In contrast, studies relying on self-reported outcomes showed no significant association and high heterogeneity.

### Subgroup analysis

**Figure 5:**
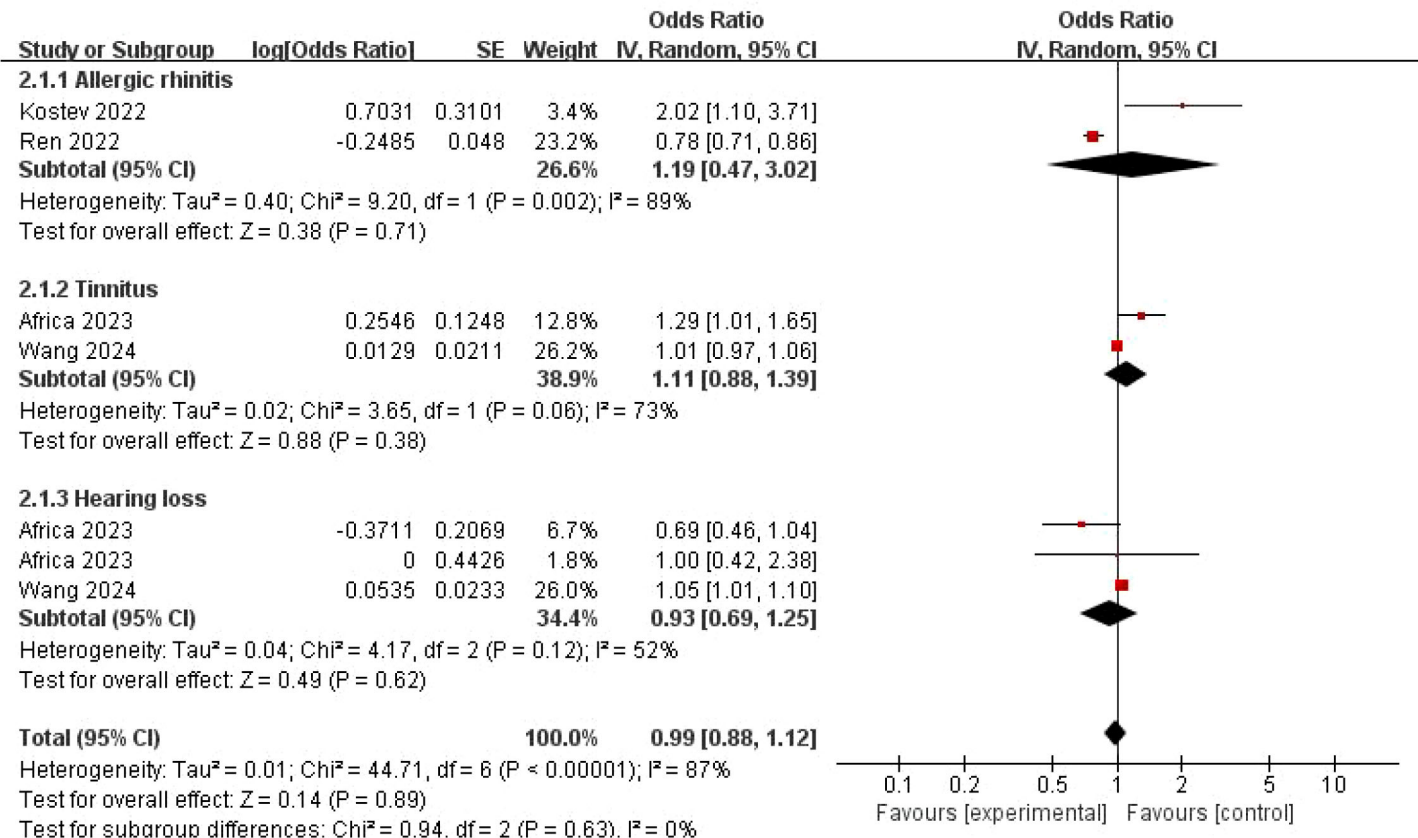
Forest map of the relationship between “three inflammations and one deafness” disease types and COVID-19.

Due to significant heterogeneity, we performed a subgroup analysis by different disease types (AR, tinnitus, and hearing loss). Analyses by specific conditions yielded mixed results: No significant association was observed for hearing loss (OR = 0.93, 95% CI: 0.69–1.25, *P* = 0.62). For AR (OR = 1.19, 95% CI: 0.47–3.02, *P* = 0.71) and tinnitus (OR = 1.11, 95% CI: 0.88–1.39, *P* = 0.38), the point estimates suggested potential positive trends, but the associations were not statistically significant, and confidence intervals were wide (**Figure 5**).

In addition, subgroup analyses were performed according to region, study design, and disease criteria. Notably, subgroup analyses revealed that the diagnostic criteria for “three inflammations and one deafness” were a key source of this heterogeneity. A strong and statistically significant association was found specifically in the subgroup where “three inflammations and one deafness” was physician-diagnosed (OR = 1.30, 95% CI: 1.08–1.56, *P* = 0.006), with no evidence of heterogeneity among these studies (*I*² = 0%). In contrast, the subgroup based on self-reported outcomes showed no significant association (OR = 0.89, 95% CI: 0.69–1.15, *P* = 0.38) and exhibited extreme heterogeneity (*I*² = 96%). Other subgroup analyses did not reveal consistent significant associations. Results varied by geographical region and were non-significant regardless of the diagnostic method for COVID-19 (physician-diagnosed or self-reported). Notably, significant residual heterogeneity persisted in most of these other subgroups (e.g., European region: *I*² = 84%; all cohort studies: *I*² = 89%) (**Table 3**).

**Table 3:** Subgroup analysis of “three inflammations and one deafness” and COVID-19.

subgroup type	*N* studies	Subgroup analysis	
		OR [95% CI] (*P*-value)	Heterogeneity—*I*^2^; *P*-value
Region			
Europe	3	0.97 [0.60, 1.57] (0.89)	84%; 0.002
Global region	1	1.29 [1.01, 1.65] (0.04)	–
Asia	1	1.01 [0.97, 1.06] (0.54)	–
Study design			
Cohort study	5	1.03 [0.85, 1.26] (0.74)	89%; <0.00001
Criteria of “three inflammations and one deafness”			
Physician diagnosis	3	1.30 [1.08, 1.56] (0.006)	0%; 0.61
Self-report	2	0.89 [0.69, 1.15] (0.38)	96%; <0.00001
Criteria of COVID-19			
Physician diagnosis	4	1.06 [0.72, 1.55] (0.78)	88%; <0.0001
Self-report	1	1.01 [0.97, 1.06] (0.54)	–

“–”: There was no heterogeneity for a single study.

## Discussion

A study had found that tinnitus and deafness could be caused by COVID-19 (17). Another study suggested that AR might be a protective factor against COVID-19 severity, but the evidence was inconclusive. COVID-19 patients with AR showed a trend toward lower hospitalization rates (OR = 0.23, 95% CI: 0.02–2.67). Furthermore, there was no statistically significant association between AR and the risk of severe disease (OR = 0.79, 95% CI: 0.52–1.18, *P* = 0.25) (18). However, these studies were reported on a single disease type. Meta-analyses were rare, lacking even an assessment of pharyngitis, otitis media, and COVID-19. Therefore, we conducted a comprehensive evaluation of the association between “three inflammations and one deafness” and COVID-19 based on five studies.

The pooled analysis using a random-effects model showed no statistically significant association between COVID-19 and “three inflammations and one deafness.” However, the biological plausibility of our epidemiological finding—that COVID-19 is associated with physician-diagnosed “three inflammations and one deafness”—can be considered in light of the established mechanism of SARS-CoV-2 cellular entry. SARS-CoV-2 is the causative agent of COVID-19. The virus comprises multiple strains, and different variants of concern exhibit significant differences in transmissibility, cellular tropism, immune evasion capacity, and pathogenicity. These differences directly contribute to variations in the severity of COVID-19. For instance, compared to early strains, the Omicron variant demonstrates stronger tropism for upper respiratory tract tissues and relatively reduced tropism for lung tissues. This aligns with clinical observations of a significantly higher reported incidence of upper respiratory symptoms such as sore throat and hoarseness in the population, alongside a relatively lower incidence of severe pneumonia. In contrast, the Delta variant is associated with higher viral loads, stronger lung tissue tropism, and worse clinical outcomes. Such variations suggest that the incidence and clinical manifestations of the “three inflammations and one deafness” focused on in this study may dynamically change depending on the dominant circulating variant.

In addition, it is well-established that SARS-CoV-2 utilizes the angiotensin-converting enzyme 2 (ACE2) receptor for host cell entry (19–21). Prior experimental studies have demonstrated the expression of ACE2 (and associated entry co-factors such as TMPRSS2) in tissues relevant to our outcomes: in the nervous system (22–25), in the upper respiratory tract including the nasal epithelium (26–28), and critically, within the cochlea (29) and middle ear (30, 31). Furthermore, SARS-CoV-2 is a recognized causative agent of viral pharyngitis (32). Therefore, the anatomical presence of the viral entry machinery in ENT tissues provides a plausible mechanistic basis that is consistent with our clinical observation of an increased risk of these complications following COVID-19.

Based on our research findings and biological plausibility, we propose two clinical practice and research recommendations. First, clinicians should be vigilant about the potential risk of otolaryngological complications when managing COVID-19 patients. Second, medications that may cause ototoxicity and other side effects should be avoided during COVID-19 treatment. Future studies should prioritize elucidating the pathophysiological mechanisms between COVID-19 and specific otolaryngological outcomes, as well as exploring other modifiable risk factors to develop comprehensive prevention strategies.

First, we explored the association between multiple common diseases in otolaryngology (AR, pharyngitis, otitis media, tinnitus, and deafness) and COVID-19, involving a wide range of diseases; second, four studies were of high quality (total score ≥ 7); third, participants with a cumulative total of over 460,000 ensured a sufficient sample size; and fourth, the age range of the participants was broad.

This study has limitations. First, the studies were all cohort studies. Moreover, we included only five studies and lacked more research analysis; second, the studies on pharyngitis and otitis media involved only one paper, respectively, lacking more data support; third, the study areas were mainly concentrated in Europe, without studies in other regions such as Oceania, Africa, and others. Fourth, the study only included English-language literature, which may have overlooked relevant non-English research.

## Conclusion

In conclusion, this meta-analysis does not provide sufficient evidence to support a statistically significant association between “three inflammations and one deafness” and COVID-19. Furthermore, the evidence synthesized in this study indicates that COVID-19 exhibits distinct association patterns with different otolaryngological diseases. The extremely high heterogeneity observed in the primary analysis suggests that these outcomes should not be conflated. Current limited evidence suggests that COVID-19 may be associated with an increased risk of AR and tinnitus, though these associations remain imprecise. No clear association was found between COVID-19 and hearing loss. These differences likely reflect varying pathological mechanisms and measurement methods. Future research should independently investigate the association between COVID-19 and each specific otolaryngological disease outcome, employing objective and standardized diagnostic tools. Additionally, efforts should be made to include multilingual databases or collaborate with multilingual research teams to minimize bias and heterogeneity.

## Data Availability

The original contributions presented in the study are included in the article/**Supplementary Material**. Further inquiries can be directed to the corresponding author.
